# The influence of the schedule and the dose of gemcitabine on the anti-tumour efficacy in experimental human cancer.

**DOI:** 10.1038/bjc.1993.285

**Published:** 1993-07

**Authors:** E. Boven, H. Schipper, C. A. Erkelens, S. A. Hatty, H. M. Pinedo

**Affiliations:** Department of Medical Oncology, Free University Hospital, Amsterdam, The Netherlands.

## Abstract

The therapeutic efficacy of gemcitabine, a new nucleoside analogue, was assessed in a variety of well-established human soft tissue sarcoma and ovarian cancer xenografts grown s.c. in nude mice. Tumour lines selected had different histological subtypes, growth rates and sensitivities to conventional cytostatic agents. The three different doses and schedules designed on the basis of a mean weight loss between 5% and 15% were i.p. injections of daily 3.5 mg kg-1 x 4, every 3 days 120 mg kg-1 x 4, and weekly 240 mg kg-1 x 2, which ultimately resulted in 19%, 10% and 4% toxic deaths, respectively. The weekly schedule induced > or = 50% growth inhibition in 2/4 soft tissue sarcoma and 4/6 ovarian cancer lines, while in three ovarian cancer lines > or = 75% growth inhibition was obtained. The anti-tumour effects of gemcitabine appeared to be similar or even better than previous data with conventional drugs tested in the same tumour lines. In comparison with the every 3 days schedule, the weekly and the daily schedule were less effective in 5/7 and 3/3 tumour lines (P < 0.001), respectively. In another experiment in three human tumour lines selected for their differential sensitivity to gemcitabine, weekly injections of 240 mg kg-1 x 6 did not result in a significant increase in the percentages of growth inhibition when compared to lower doses of 120 mg kg-1 or 60 mg kg-1 in the same schedule. However, the 240 mg kg-1 weekly x 6 schedule showed superior effects in 2/3 tumour lines in comparison with the same dose given every 2 weeks x 3 (P < 0.05). The preclinical activity of gemcitabine suggests that the drug can induce responses in soft tissue sarcoma and ovarian cancer patients. Our results further indicate that clinical trials of gemcitabine in solid tumour types should be designed on the basis of a schedule rather than a dose dependence.


Br. J. Cancer (1993), 68, 52–56 © Macmillan Press Ltd., 1993

### The influence of the schedule and the dose of gemcitabine on the anti-tumour efficacy in experimental human cancer

E. Boven[1], H. Schipper[1], C.A.M. Erkelens[2], S.A. Hatty[3] & H.M. Pinedo[1]

[1]*Department of Medical Oncology and* [2]*Experimental Animal Laboratory, Free University Hospital, De Boelelaan 1117, 1081 HV Amsterdam, The Netherlands and* [3]*Lilly Research Centre Limited, Windlesham, Surrey, UK.*

**Summary** The therapeutic efficacy of gemcitabine, a new nucleoside analogue, was assessed in a variety of well-established human soft tissue sarcoma and ovarian cancer xenografts grown s.c. in nude mice. Tumour lines selected had different histological subtypes, growth rates and sensitivities to conventional cytostatic agents. The three different doses and schedules designed on the basis of a mean weight loss between 5% and 15% were i.p. injections of daily 3.5 mg kg$^{-1}$ × 4, every 3 days 120 mg kg$^{-1}$ × 4, and weekly 240 mg kg$^{-1}$ × 2, which ultimately resulted in 19%, 10% and 4% toxic deaths, respectively. The weekly schedule induced ⩾ 50% growth inhibition in 2/4 soft tissue sarcoma and 4/6 ovarian cancer lines, while in three ovarian cancer lines ⩾ 75% growth inhibition was obtained. The anti-tumour effects of gemcitabine appeared to be similar or even better than previous data with conventional drugs tested in the same tumour lines. In comparison with the every 3 days schedule, the weekly and the daily schedule were less effective in 5/7 and 3/3 tumour lines ($P<0.001$), respectively. In another experiment in three human tumour lines selected for their differential sensitivity to gemcitabine, weekly injections of 240 mg kg$^{-1}$ × 6 did not result in a significant increase in the percentages of growth inhibition when compared to lower doses of 120 mg kg$^{-1}$ or 60 mg kg$^{-1}$ in the same schedule. However, the 240 mg kg$^{-1}$ weekly × 6 schedule showed superior effects in 2/3 tumour lines in comparison with the same dose given every 2 weeks × 3 ($P<0.05$). The preclinical activity of gemcitabine suggests that the drug can induce responses in soft tissue sarcoma and ovarian cancer patients. Our results further indicate that clinical trials of gemcitabine in solid tumour types should be designed on the basis of a schedule rather than a dose dependence.

In the search for new anti-cancer agents, gemcitabine (2′,2′-difluorodeoxycytidine, dFdC) has recently emerged from the preclinical drug development stage as a promising candidate for the treatment of non-haemotological malignancies. Gemcitabine is a new antimetabolite active against human leukaemic cell lines *in vitro* and a number of solid murine and human tumours in mice (Hertel *et al.*, 1990; Braakhuis *et al.*, 1991). The preclinical activity of gemcitabine was more pronounced than that of the nucleoside analogue 1-β-D-arabinofuranosylcytosine (ara-C), a drug commonly used in the treatment of adult acute leukaemia. Both drugs inhibit cellular proliferation in S phase and cause cells to accumulate in the $G_1$-S phase (Hertel *et al.*, 1990). The apparent similarities in the molecular structures and the reversal of cytotoxicity by deoxycytidine led to comparative studies on the metabolic and pharmacokinetic characteristics of gemcitabine and ara-C (Heinemann *et al.*, 1988). It was found in Chinese hamster ovary cells, that the cellular concentration of the 5′-triphosphate of gemcitabine (dFdCTP) was 20-fold greater than that observed for ara-CTP at equimolar concentrations of the drugs. These differences in cellular accumulation of the respective triphosphates were due to an increased membrane transport, a higher deoxycytidine kinase affinity, and a longer retention of the intracellular 5′-triphosphate for gemcitabine relative to ara-C. The favourable characteristics of gemcitabine were the reason to introduce the drug into clinical trials (Abbruzzese *et al.*, 1991; Grunewald *et al.*, 1992).

The preclinical *in vivo* analysis of the anti-tumour efficacy of gemcitabine was first carried out in a variety of the well-known murine and human tumour systems (Hertel *et al.*, 1990). On the basis of a significant reduction in growth and cures in xenografts derived from squamous cell carcinoma tissue of the head and neck region, Braakhuis *et al.* (1991) suggested the drug of potential value in the treatment of head and neck cancer patients. In order to obtain further insight into the possible activity of gemcitabine against other malignancies, we extended the preclinical *in vivo* anti-tumour screening of gemcitabine in human tumour xenografts selected from two panels of well-defined human soft tissue sarcoma and ovarian cancer lines. In the experiments, we put emphasis on the influence of the schedule and the dose of gemcitabine to reach optimal growth inhibition.

### Materials and methods

### *Animals and tumour lines*

Female NMRI/Cpb nude mice (Harlan Cpb, Zeist, The Netherlands) were maintained in filter-top cages under controlled atmospheric conditions. Cages, covers, food, bedding and water were sterilised and changed once a week. Animal handling was done in a laminar down-flow hood.

Tumour lines were selected from a panel of ten human soft tissue xenografts and 15 human ovarian cancer xenografts on the basis of differences in histological subtype and growth rate, as well as their sensitivity to equitoxic doses of conventional cytostatic agents. Most characteristics mentioned in Table I have been described previously (Winograd *et al.*, 1987; Boven *et al.*, 1989; Molthoff *et al.*, 1991). Tumour lines were maintained by serial subcutaneous (s.c.) transplantation of tumour fragments of 2–3 mm in diameter in both flanks of 8- to 10-week-old animals.

### *Treatment and evaluation*

Gemcitabine (Lilly Research Centre Limited) was provided as a powder and was dissolved in NaCl 0.9% to reach a final concentration of 10 mg ml$^{-1}$. In the first step of the experiment the drug was injected i.p. in various schedules to determine equitoxic doses. At these doses, the mice were required to lose between 5% and 15% of the initial weight within 2 weeks after the first injection. The equitoxic doses were assessed in non-tumour-bearing mice first and adjusted in tumour-bearing mice, if indicated. Thereafter, the efficacy of gemcitabine was determined in tumour-bearing mice with the use of three different doses and schedules.

Xenografts were measured once or twice (for A2780) a week in three dimensions with a vernier caliper by the same observer. The volume was calculated by the equation length × width × height × 0.5, and expressed in mm$^3$. At the

Correspondence: E. Boven.
Received 27 August 1992; and in revised form 1 March 1993.

**Table I** Characteristics of human tumour lines grown s.c. in nude mice

| *Tumour line* | *Histology* | *TD*[a] | *CDDP*[c] | *CTX* | *DOX* | *GEM* |
|---|---|---|---|---|---|---|
| | | | *Growth inhibition*[b] | | | |
| *Soft tissue sarcoma* | | | | | | |
| S.Ho | Malignant fibrous histiocytoma | 16 | n.d.[d] | ++ | − | + |
| S.La(C) | Malignant fibrous histiocytoma | 11 | n.d. | − | − | + |
| S.Hu | Leiomyosarcoma | 12 | n.d. | − | − | − |
| S.To | Synovial cell sarcoma | 13 | n.d. | − | − | − |
| *Ovarian cancer* | | | | | | |
| Ov.Pe | Moderately differentiated mucinous | 8 | − | + | + | ++ |
| Ov.He | Moderately differentiated mucinous | 9 | − | − | + | + |
| OVCAR-3 | Poorly differentiated serous | 8 | ++ | + | − | ++ |
| A2780 | Undifferentiated | 3.5 | + | ++ | ++ | ++ |
| FKo | Moderately differentiated serous | 12 | − | − | − | − |
| Ov.Ri(C) | Moderately differentiated serous | 11 | ++ | + | + | − |

[a]Mean volume doubling time in days. [b]< 50% (−); ⩾ 50% < 75% (+); ⩾ 75% (++). [c]CDDP, cisplatin 5 mg kg$^{-1}$ i.v. q7dx2; CTX, cyclophosphamide 150 mg kg$^{-1}$ i.p. q14dx2; DOX, doxorubicin 8 mg kg$^{-1}$ i.v. q7dx2; gemcitabine 240 mg kg$^{-1}$ i.p. q7dx2. [d]n.d., not determined.

start of treatment (day 0), groups of 5–7 tumour-bearing mice were formed to provide 8–14 tumours with a mean volume of 50–150 mm$^3$ in each group. Deaths occurring within 2 weeks after the final injection were considered toxic deaths; these animals were excluded from the study.

For evaluation of drug efficacy, the tumour volumes were converted to values related to the initial tumour volume. This relative tumour volume was expressed by the formula $V_T/V_0$, where $V_T$ is the value at any given day and $V_0$ the volume at the start of treatment. The ratio of the mean relative volume of treated tumours over that of control tumours multiplied by 100% (T/C%) was assessed on each day of measurement. From the lowest T/C% obtained within 5 weeks after the last day of injection growth inhibition (100%−T/C%) was calculated to express drug efficacy. Complete remissions were defined as the total disappearance of tumours without regrowth in the following month. Differences of significance between the anti-tumour effects of particular treatment regimens were determined by means of Student's *t*-test.

### Results

### *Doses and schedules*

The equitoxic doses of gemcitabine were based on the induction of a mean weight loss between 5% and 15% and were determined for daily × 5, every 3 days × 4, and weekly × 2 i.p. injections in non-tumour-bearing animals first. Starting doses were derived from previous experiments carried out by other investigators (Hertel *et al.*, 1990; Braakhuis *et al.*, 1991). Doses of 10 mg kg$^{-1}$, 5 mg kg$^{-1}$, and 2.5 mg kg$^{-1}$ were administered for daily injections, but proved to be too toxic (mean weight loss >20%) for doses of ⩾5 mg kg$^{-1}$. Further adjustment of the dose in steps of 4 mg kg$^{-1}$, 3.5 mg kg$^{-1}$ and 3 mg kg$^{-1}$ led to a mean weight loss of >20%, 11% (s.d. ± 1%), and 7% (s.d. ± 3%), respectively. The schedule of 3.5 mg kg$^{-1}$ i.p. daily × 5 was selected, but a mean weight loss >20% in tumour-bearing animals necessitated a reduction of 1 day. For the every 3 days × 4 schedule a dose of 120 mg kg$^{-1}$ was used, which was determined to be the optimal dose in the same strain of mice by Braakhuis *et al.* (1991). In fact, increasing this dose to 140 mg kg$^{-1}$ in non-tumour-bearing animals resulted in a mean weight loss >20%. For weekly × 2 injections a dose range of 120 mg kg$^{-1}$ with increases in steps of 40 mg kg$^{-1}$ up to 280 mg kg$^{-1}$ was tested. Doses of 240 mg kg$^{-1}$ and 280 mg kg$^{-1}$ led to a mean weight loss of 6% (s.d. ± 7%) and 13% (s.d. ± 3%), respectively, while lower doses did not induce loss of weight. Because of additional weight loss to be expected in tumour-bearing animals, the weekly dose of 240 mg kg$^{-1}$ was selected.

In the treatment experiments animals were weighed only at the time of the injections or the weekly tumour measurements. Mean weight loss was calculated within 2 weeks after the first injection in non-lethal mice (Table II). Except for toxic deaths, recovery from weight loss was rapid and invariably reversible within 1 week after treatment. Thus, mean weight loss for the weekly schedule depicted in Table II does not reflect the maximum weight loss to be expected, if

**Table II** Growth inhibition induced by gemcitabine administered i.p. at the maximum tolerated dose in various schedules

| *Tumour line* | *3.5 mg kg$^{-1}$ daily × 4* GI% | (day)[a] | WL[b] | TD[c] | *120 mg kg$^{-1}$ every 3 days × 4* GI% | (day) | WL | TD | *240 mg kg$^{-1}$ weekly × 2* GI% | (day) | WL | TD |
|---|---|---|---|---|---|---|---|---|---|---|---|---|
| *Soft tissue* | | | | | | | | | | | | |
| S.Ho | | | | | | | | | 52%[d] | (13) | 2% ± 2% | 2/6 |
| S.La(C) | 47%[d] | (28) | 5% ± 2% | 0/6 | 86%[d,e] | (28) | 4% ± 2% | 0.6 | | | | |
| S.La(C) | 23% | (31) | 26% ± 2% | 4/6 | 89%[d,e] | (31) | 9% ± 6% | 0/6 | 74%[d] | (31) | 3% ± 4% | 0/6 |
| S.Hu | | | | | | | | | 27% | (35) | 3% ± 7% | 0/6 |
| S.Hu | 54%[d] | (32) | 14% ± 5% | 1/7 | 89%[d,e,f] | (32) | 7% ± 6% | 1/7 | 49%[d] | (32) | 5% ± 6% | 0/7 |
| S.To | | | | | | | | | 10% | (36) | 0% ± 3% | 0/6 |
| S.To | | | | | 17% | (20) | 13% ± 14% | 0/6 | 23% | (20) | 0% ± 2% | 0/6 |
| *Ovary* | | | | | | | | | | | | |
| Ov.Pe | | | | | | | | | 82%[d] | (30) | 5% ± 3% | 0/6 |
| Ov.Pe | 61%[d] | (33) | 9% ± 6% | 0/7 | 77%[d,e,f] | (33) | 9% ± 7% | 0.7 | 70%[d] | (33) | 0% ± 3% | 0/7 |
| Ov.He | | | | | 84%[d,f] | (35) | 15% ± 12% | 3/6 | 64%[d] | (28) | 5% ± 3% | 0/6 |
| OVCAR-3 | | | | | 98%[d,f] | (27) | 14% ± 10% | 1/6 | 92%[d] | (20) | 0% ± 4% | 0/6 |
| A2780 | | | | | 99%[d,f] | (27) | 19% ± 8% | 0/6 | 98%[d] | (18) | 16% ± 6% | 0/6 |
| FKo | | | | | | | | | 11% | (39) | 6% ± 5% | 1/6 |
| Ov.Ri(C) | | | | | | | | | 36% | (28) | 1% ± 3% | 0/6 |

[a]GI, growth inhibition (%) and optimal day of measurement. [b]WL, weight loss (% ± s.d.) within 2 weeks after the first injection. [c]TD, toxic death within 2 weeks after the final injection. [d]Significantly different from control tumours, $P < 0.001$. [e]120 mg kg$^{-1}$ i.p. given every 3 days × 4 shows significantly superior growth inhibition to 3.5 mg kg$^{-1}$ i.p. daily × 4, $P < 0.001$. [f]120 mg kg$^{-1}$ i.p. given every 3 days × 4 shows significantly superior growth inhibition to 240 mg kg$^{-1}$ i.p. weekly × 2, $P < 0.001$.

animals had been weighed more often. As an example, mice bearing A2780 xenografts were weighed twice-a-week and mean weight loss recorded was 16% (s.d. ± 6%), which illustrates the equal toxicity of the weekly schedule. For the every 3 days schedule and the daily schedule mean weight loss varied for the mice bearing different human tumour lines and was, in general, in the range between 5% and 15% of the initial weight. With reference to toxic deaths (Table II) the weekly schedule appeared to be the least toxic as 4% of animals died, followed by 10% toxic deaths in the every 3 days schedule. Using the daily schedule, 19% of animals died from toxicity.

### *Influence of schedule*

Gemcitabine at equitoxic doses was administered in three schedules (Table II). The weekly schedule of 240 mg $kg^{-1} \times 2$ was studied in all human tumour lines. Growth inhibition of $\geqslant$50% was obtained in 2/4 soft tissue sarcoma xenografts and 4/6 ovarian cancer xenografts. In Ov.Pe, OVCAR-3 and A2780 xenografts $\geqslant$75% inhibition of growth could be measured. In comparison with previous experiments with conventional cytostatic agents (Table I), gemcitabine appeared slightly more effective than doxorubicin in S.Ho xenografts and than doxorubicin and cyclophosphamide in S.La(C) xenografts. In Ov.Pe xenografts, gemcitabine was superior to cisplatin, cyclophosphamide and doxorubicin, but the reverse was observed in Ov.Ri(C) xenografts. The percentages of growth inhibition of gemcitabine calculated in Ov.He, OVCAR-3 and A2780 xenografts were similar to or better than the data for the three conventional agents. Against S.Hu, S.To and FKo xenografts the clinically known compounds were inactive, as was gemcitabine.

The weekly schedule of gemcitabine was compared to the every 3 days schedule of 120 mg $kg^{-1} \times 4$ in seven human tumour lines (Table II). In five of these, the every 3 days schedule was significantly more effective ($P<0.001$). With this schedule, 4/11 complete remissions could be obtained in A2780 xenografts. The anti-tumour effects reached with the weekly schedule were not significantly different from the extent of growth inhibition observed after the administration of 3.5 mg $kg^{-1}$ daily $\times$ 4. Again, the every 3 days schedule was significantly more effective than the daily schedule ($P<0.001$) in S.La(C), S.Hu and Ov.Pe xenografts. Figure 1

**Figure 1** Treatment results of gemcitabine administered i.p. at the maximum tolerated dose of 240 mg $kg^{-1}$ weekly $\times$ 2, 120 mg $kg^{-1}$ every 3 days $\times$ 4, or 3.5 mg $kg^{-1}$ daily $\times$ 4 (- - - -) in three human tumour xenografts as compared to control tumours (———). The relative tumour volume is the tumour volume at any given day $V_T$/the volume at the start of treatment $V_0$. The graphs were drawn from the mean ( ± s.e.m.) of the relative tumour volumes.

visualises the superior efficacy of the every 3 days schedule compared to the weekly and the daily schedule in S.La(C), S.Hu and Ov.Pe xenografts.

### *Influence of dose*

Three human tumour lines with a variable degree of sensitivity to the weekly × 2 schedule of gemcitabine, S.La(C), S.Hu and Ov.Pe, were selected to study the presence of a possible relationship between the dose and the response to the drug. Tumour-bearing mice were treated for 6 weeks with weekly injections of 240 mg $kg^{-1}$, 120 mg $kg^{-1}$ or 60 mg $kg^{-1}$ and another group of animals was treated with 2-weekly injections of 240 mg $kg^{-1}$. In Figure 2 it is shown, that gemcitabine resulted in the reduction of tumour volume in S.La(C) xenografts, in stabilisation of tumour growth in Ov.Pe xenografts, while in S.Hu xenografts limited growth delay was obtained. The anti-tumour effects of the various doses expressed in percentages of growth inhibition (Table III) were not greatly different. The 240 mg $kg^{-1}$ weekly × 6 schedule showed superior effects only in S.La(C) and S.Hu xenografts when compared to the same dose given every 2 weeks × 3, but the difference hardly reached the level of significance ($P<0.05$). However, in S.La(C) xenografts the number of complete remissions was highest for the 240 mg $kg^{-1}$ × 6 schedule (9/10), followed by 120 mg $kg^{-1}$ × 6 and 60 mg $kg^{-1}$ × 6 (in both schedules 6/10) and the 240 mg $kg^{-1}$ 2-weekly × 3 schedule (4/8).

### Discussion

For ara-C in the treatment of adult acute leukaemia patients it has been recognised, that drug efficacy is related to the schedule of administration, where a continuous infusion will induce a higher response rate than daily conventional doses over periods of 5–10 days (Freireich, 1987). Gemcitabine also shows a schedule dependence. In our experiments we demonstrated, that the slightly more toxic daily schedule was less effective than the every 3 days schedule. A similar experience was reported by Hertel *et al.* (1990) in L1210 leukaemia. In addition we found, that the longer interval of 1 week between the injections will again result in a lower degree of growth inhibition. The clinical relevance of this finding is not yet known. In the various phase II trials presently underway a weekly schedule is being used consisting of treatment for 3 weeks followed by 1 week's rest (Lund *et al.*, 1993). Similar to the large differences in the administered doses per schedule in mice, patients can tolerate a considerably higher weekly dose of gemcitabine (the dose recommended for phase II trials is 1000 mg $m^{-2}$) than a daily dose of the drug (recommended dose 9 mg $m^{-2}$), while in a 2-weekly schedule a dose of 4560 mg $m^{-2}$ was well tolerated.

The low dose-response relationship for gemcitabine found in our human tumour lines may be explained by the cellular pharmacology of the drug. Phosphorylation by deoxycytidine kinase is required to induce cytotoxicity upon incorporation of dFdCTP into DNA. Inactivation of phosphorylated gemcitabine is caused by deamination to difluorouridine, a reaction catalysed by deoxycytidine deaminase (Heinemann *et al.*, 1988; Gandhi & Plunkett, 1990). In phase I clinical trials,

**Figure 2** Treatment results of gemcitabine administered i.p. at the maximum tolerated dose of 240 mg $kg^{-1}$ weekly × 6 (▼), and lower doses of 120 mg $kg^{-1}$ weekly × 6 (■), 60 mg $kg^{-1}$ weekly × 6 (▲), or 240 mg $kg^{-1}$ 2-weekly × 3 (◆), as compared to control tumours (●). The relative tumour volume is the tumour volume at any given day $V_T$/the volume at the start of treatment $V_0$. The graphs were drawn from the mean of the relative tumour volumes.

**Table III** Growth inhibition induced by gemcitabine administered i.p. weekly or 2-weekly in various doses

| *Tumour line* | *Day*[a] | *60 mg kg⁻¹ weekly × 6* | *120 mg kg⁻¹ weekly × 6* | *240 mg kg⁻¹ weekly × 6* | *240 mg kg⁻¹ 2-weekly × 3* |
|---|---|---|---|---|---|
| S.La(C) | 49 | 96.8% | 99.4% | 99.8%[b] | 83% |
| S.Hu | 49 | 55% | 65% | 65%[b] | 42% |
| Ov.Pe | 50 | 66% | 72% | 72% | 65% |

[a]Day of measurement. [b]240 mg $kg^{-1}$ i.p. weekly × 6 shows significantly superior growth inhibition to 240 mg $kg^{-1}$ i.p. 2-weekly × 3, $P<0.05$.

Grunewald et al. (1990, 1991, 1992) have shown that the accumulation rate of dFdCTP in both leukaemia and mononuclear cells is saturated at certain plasma or intracellular drug levels. At higher gemcitabine doses, no further increase or even a decreased value of dFdCTP could be measured. Our experiments suggest, that the phosphorylation of gemcitabine in tumour cells is also a saturable process. Further pharmacodynamic analysis of the intracellular metabolism of gemcitabine will clarify the precise mechanism of action and the variation in cytotoxicity between tumour cells.

If a relationship exists between the ability to accumulate and retain dFdCTP and the response, as has been demonstrated for high-dose continuous infusion of ara-C (Estey et al., 1990), it is anticipated that in S.La(C) xenografts a higher area under the concentration-times-time curve (AUC) for dFdCTP can be reached when compared to S.Hu xenografts. In patients, the AUC of dFdCTP in mononuclear cells indeed augmented with prolonged administration of gemcitabine at a dose maintaining maximal dFdCTP accumulation (Grunewald et al., 1991). In tumour cells exposed to gemcitabine *in vitro*, dFdCTP accumulation was clearly demonstrated to be time dependent (Ruiz van Haperen et al., 1991). Whether longer infusion periods will result in higher anti-tumour efficacy rather than increased side-effects in patients has yet to be determined.

In our laboratory we have found a good correlation between clinical data from phase II trials and the chemosensitivity of the panels of human soft tissue sarcoma xenografts and human ovarian cancer xenografts, both for conventional cytostatic agents and their analogues (Boven et al., 1985; Winograd et al., 1987; Boven, 1988; Boven et al., 1989; Boven et al., 1990; Boven, 1991). The validity of the xenograft model has not yet been proven for all classes of antitumour agents, such as antimetabolites. On one hand, pharmacological differences between mouse and man may specifically be responsible for a negative correlation. As examples, mice can tolerate much lower doses of methotrexate and 5-fluorouracil relative to maximum doses in patients, which may be the reason that these drugs have low or no efficacy in head and neck cancer xenografts (Braakhuis et al., 1983) and colon cancer xenografts (Mattern et al., 1988), respectively. On the other hand, a higher proportion of rapidly proliferating tumour cells in the log-phase of growth in xenografts as compared to patients' tumours may theoretically result in an increased susceptibility to the cytotoxicity of other antimetabolites, as may be the case for gemcitabine. However it appears, that the xenograft model can indeed predict clinical activity of gemcitabine in particular solid tumour types. Objective responses have been noted in a variety of malignancies, including non-small cell lung cancer, ovarian cancer and breast cancer patients (Lund et al., 1993).

In conclusion, gemcitabine is a new nucleoside analogue with a unique mechanism of action, which should be investigated further for a rational design of clinical trials. Preclinical analysis of the anti-tumour activity against human tumour xenografts suggests, that the drug may be effective in soft tissue sarcoma and ovarian cancer patients.

### References

ABBRUZZESE, J.L., GRUNEWALD, R., WEEKS, E.A., GRAVEL, D., ADAMS, T., NOWAK, B., MINEISHI, S., TARASSOF, P., SATTERLEE, W., RABER, M.N. & PLUNKETT, W. (1991). A phase I clinical, plasma, and cellular pharmacology study of gemcitabine. *J. Clin. Oncol.*, **9**, 491–498.

BOVEN, E. (1988). Conventional agents in human ovarian cancer xenografts. In *Human Tumour Xenografts in Anticancer Drug Development*, Winograd, B., Peckham, M.J. & Pinedo, H.M. (eds) pp. 33–35. Springer-Verlag: Berlin-Heidelberg.

BOVEN, E. (1991). Analogs of conventional agents. In *The Nude Mouse in Oncology Research*, Boven, E. & Winograd, B. (eds) pp. 185–198. CRC Press Inc.: Boca Raton, Fla.

BOVEN, E., CALAME, J.J., MOLTHOFF, C.F.M. & PINEDO, H.M. (1989). Characterization and chemotherapy of human soft tissue sarcoma lines grown in nude mice. *Strahlenther. Onkol.*, **165**, 538–539.

BOVEN, E., NAUTA, M.M., SCHLUPER, H.M.M., ELFERINK, F., VAN DER VIJGH, W.J.F. & PINEDO, H.M. (1985). Secondary screening of platinum compounds in human ovarian cancer xenografts in nude mice. *Eur. J. Cancer Clin. Oncol.*, **21**, 1253–1260.

BOVEN, E., SCHLUPER, H.M.M., ERKELENS, C.A.M. & PINEDO, H.M. (1990). Doxorubicin compared with related compounds in a nude mouse model for human ovarian cancer. *Eur. J. Cancer*, **26**, 983–986.

BRAAKHUIS, B.J.M, SCHOEVERS, E.J., HEINERMAN, E.C.M., SNEEUWLOPER, G. & SNOW, G.B. (1983). Chemotherapy of human head and neck cancer xenografts with three clinically active drugs: *cis*-platinum, bleomycin and methotrexate. *Br. J. Cancer*, **48**, 711–716.

BRAAKHUIS, B.J.M., VAN DONGEN, G.A.M.S., VERMORKEN, J.B. & SNOW, G.B. (1991). Preclinical *in vivo* activity of 2',2'-difluorodeoxycytidine (gemcitabine) against human head and neck cancer. *Cancer Res.*, **51**, 211–214.

ESTEY, E.H., KEATING, M.J., MCCREDIE, K.B., FREIREICH, E.J. & PLUNKETT, W. (1990). Cellular ara-CTP pharmacokinetics, response, and karyotype in newly diagnosed acute myelogenous leukemia. *Leukemia (Baltimore)*, **4**, 95–99.

FREIREICH, E.J. (1987). Arabinosyl cytosine: a 20-year update. *J. Clin. Oncol.*, **5**, 523–524.

GANDHI, V. & PLUNKETT, W. (1990). Modulatory activity of 2',2'-difluorodeoxycytidine on the phosphorylation and cytotoxicity of arabinosyl nucleosides. *Cancer Res.*, **50**, 3675–3680.

GRUNEWALD, R., ABBRUZZESE, J., TARASSOFF, P. & PLUNKETT, W. (1991). Saturation of 2'-2'-difluorodeoxycytidine 5'-triphosphate accumulation by mononuclear cells during a phase I trial of gemcitabine. *Cancer Chemother. Pharmacol.*, **27**, 258–262.

GRUNEWALD, R., KANTARJIAN, H., DU, M., FAUCHER, K., TARASSOFF, P. & PLUNKETT, W. (1992). Gemcitabine in leukemia: a phase I clinical, plasma, and cellular pharmacology study. *J. Clin. Oncol.*, **10**, 406–413.

GRUNEWALD, R., KANTARJIAN, H., KEATING, M.J., ABBRUZZESE, J., TARASSOF, P. & PLUNKETT, W. (1990). Pharmacologically directed design of the dose rate and schedule of 2'-2'-difluorodeoxycytidine (gemcitabine) administration in leukemia. *Cancer Res.*, **50**, 6823–6826.

HEINEMANN, V., HERTEL, L.W., GRINDEY, G.B. & PLUNKETT, W. (1988). Comparison of the cellular pharmacokinetics and toxicity of 2'-2'-difluorodeoxycytidine and 1-β-D-arabino-furanosylcytosine. *Cancer Res.*, **48**, 4024–4031.

HERTEL, L.W., BODER, G.B., KROIN, J.S., RINZEL, S.M., POORE, G.A., TODD, G.C. & GRINDEY, G.B. (1990). Evaluation of the antitumor activity of gemcitabine (2'-2'-difluoro-2'-deoxycytidine). *Cancer Res.*, **50**, 4417–4422.

LUND, B., KRISTJANSEN, P.E.G. & HANSEN, M.H. (1993). Clinical and preclinical activity of 2',2'-difluorodeoxycytidine (gemcitabine). *Cancer Treat. Rev.*, **19**, 45–55.

MATTERN, J., BAK, M., HAHN, E.W. & VOLM, M. (1988). Human tumor xenografts as model for drug testing. *Cancer Metast. Rev.*, **7**, 263–284.

MOLTHOFF, C.F.M., CALAME, J.J., PINEDO, H.M. & BOVEN, E. (1991). Human ovarian cancer xenografts in nude mice: characterization and analysis of antigen expression. *Int. J. Cancer*, **47**, 72–79.

RUIZ VAN HAPEREN, V.W.T., VEERMAN, G., NOORDHUIS, P., VERMORKEN, J.B. & PETERS, G.J. (1991). Concentration and time dependent growth inhibition and metabolism in vitro by 2'-2'-difluoro-deoxycytidine (gemcitabine). *Adv. Exp. Med. Biol.*, **309A**, 57–60.

WINOGRAD, B., BOVEN, E., LOBBEZOO, M.W. & PINEDO, H.M. (1987). Human tumor xenografts in the nude mouse and their value as test models in anticancer drug development. *In Vivo*, **1**, 1–14.


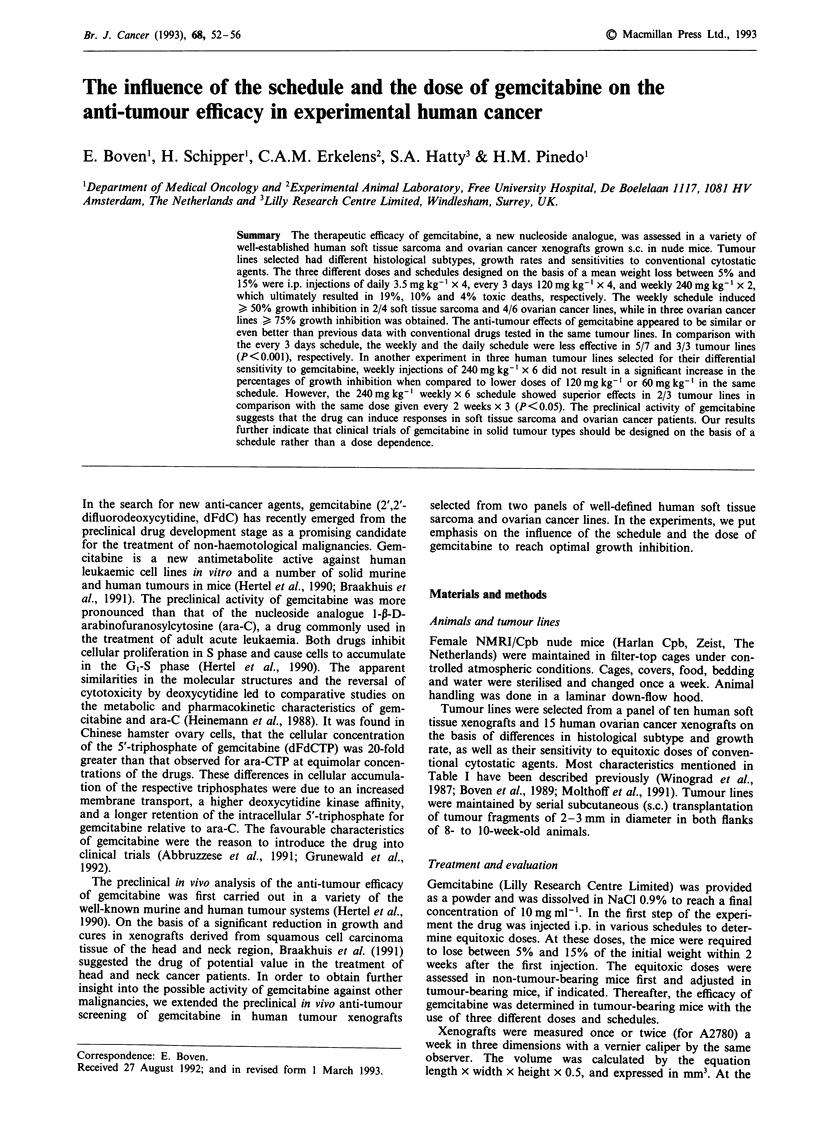

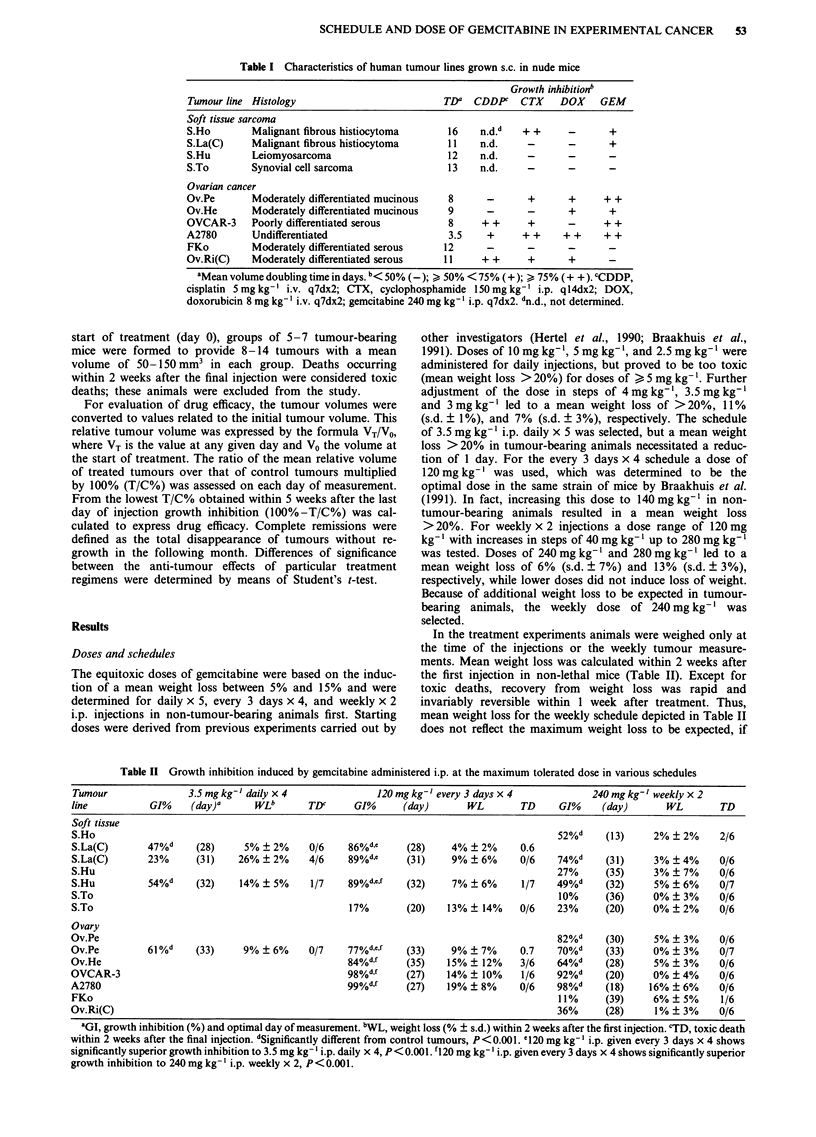

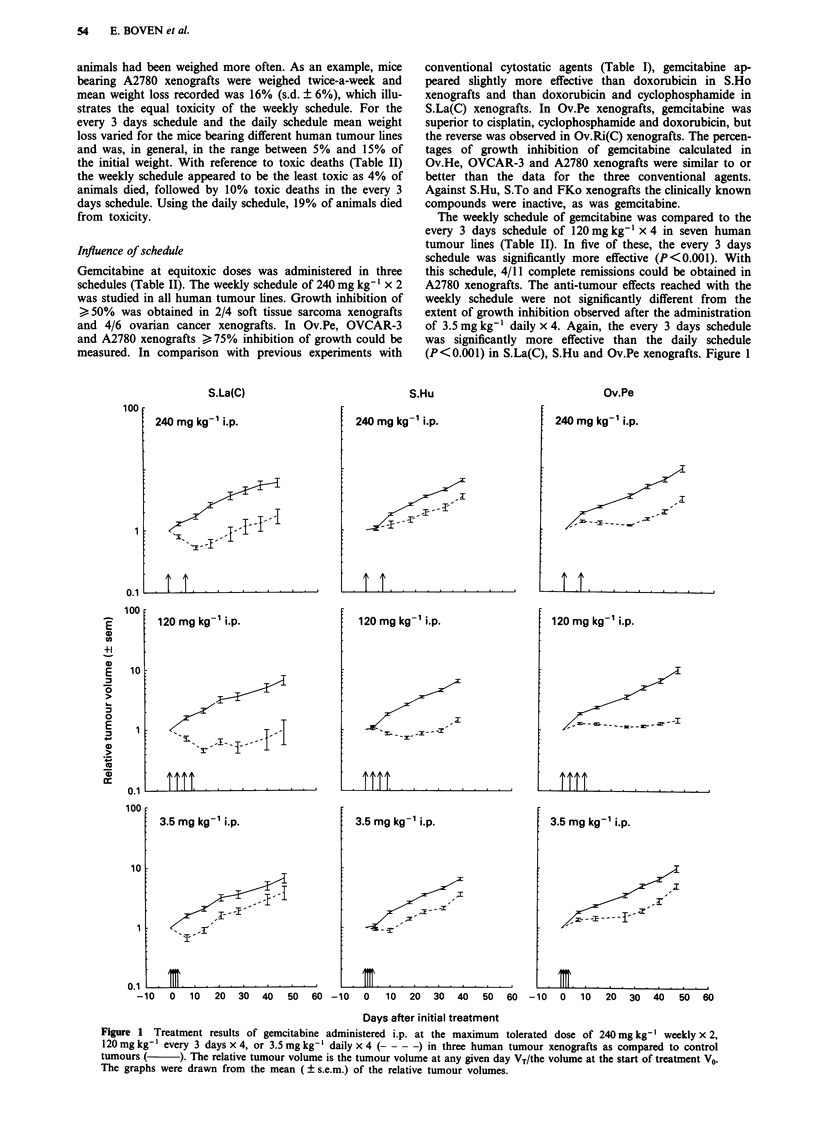

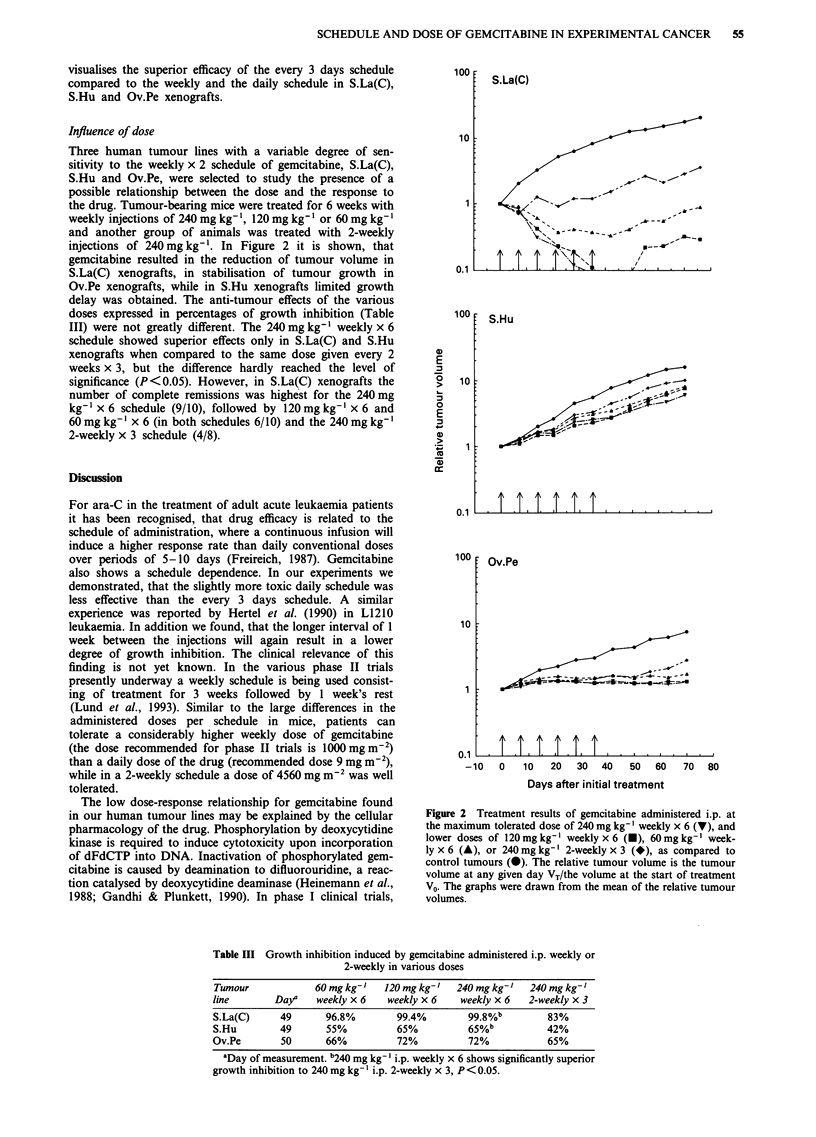

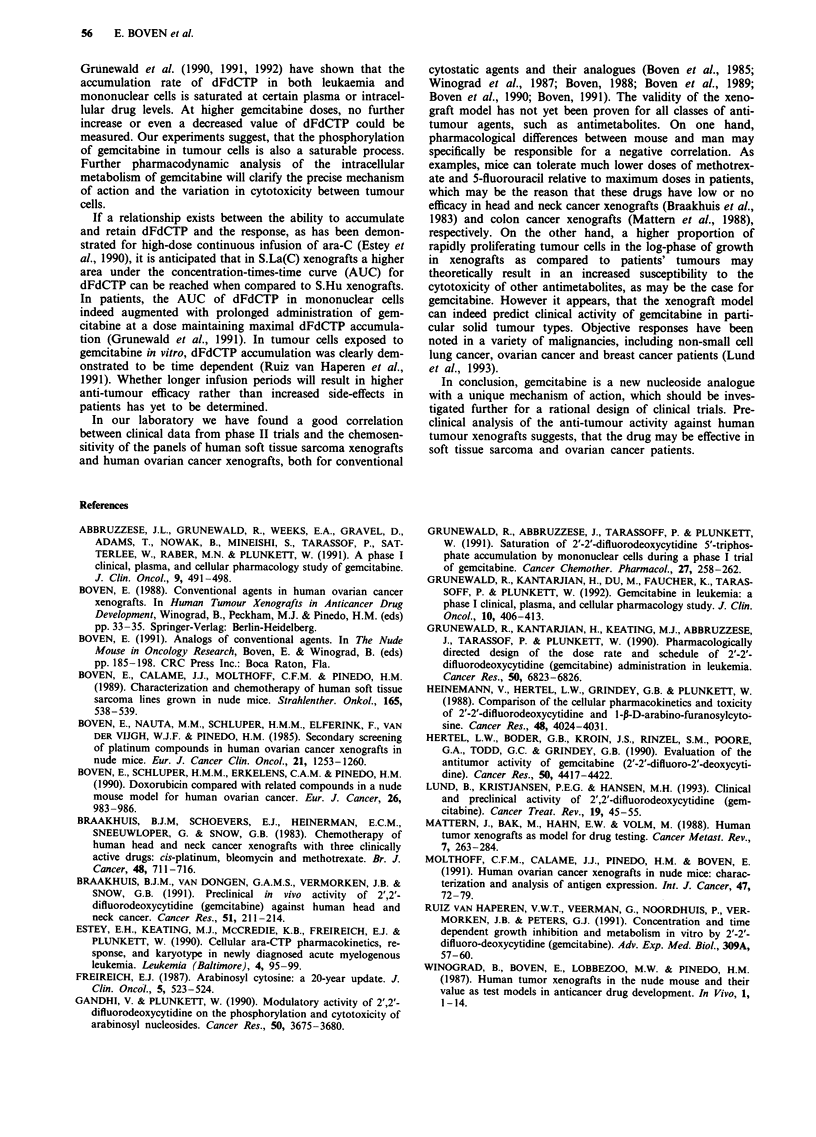


## References

[OCR_00600] Abbruzzese J. L., Grunewald R., Weeks E. A., Gravel D., Adams T., Nowak B., Mineishi S., Tarassoff P., Satterlee W., Raber M. N. (1991). A phase I clinical, plasma, and cellular pharmacology study of gemcitabine.. J Clin Oncol.

[OCR_00615] Boven E., Calame J. J., Molthoff C. F., Pinedo H. M. (1989). Characterization and chemotherapy of human soft tissue sarcoma (STS) lines grown in nude mice.. Strahlenther Onkol.

[OCR_00621] Boven E., Nauta M. M., Schluper H. M., Elferink F., van der Vijgh W. J., Pinedo H. M. (1985). Secondary screening of platinum compounds in human ovarian cancer xenografts in nude mice.. Eur J Cancer Clin Oncol.

[OCR_00627] Boven E., Schlüper H. M., Erkelens C. A., Pinedo H. M. (1990). Doxorubicin compared with related compounds in a nude mouse model for human ovarian cancer.. Eur J Cancer.

[OCR_00633] Braakhuis B. J., Schoevers E. J., Heinerman E. C., Sneeuwloper G., Snow G. B. (1983). Chemotherapy of human head and neck cancer xenografts with three clinically active drugs: cis-platinum, bleomycin and methotrexate.. Br J Cancer.

[OCR_00640] Braakhuis B. J., van Dongen G. A., Vermorken J. B., Snow G. B. (1991). Preclinical in vivo activity of 2',2'-difluorodeoxycytidine (Gemcitabine) against human head and neck cancer.. Cancer Res.

[OCR_00646] Estey E. H., Keating M. J., McCredie K. B., Freireich E. J., Plunkett W. (1990). Cellular ara-CTP pharmacokinetics, response, and karyotype in newly diagnosed acute myelogenous leukemia.. Leukemia.

[OCR_00652] Freireich E. J. (1987). Arabinosyl cytosine: a 20-year update.. J Clin Oncol.

[OCR_00656] Gandhi V., Plunkett W. (1990). Modulatory activity of 2',2'-difluorodeoxycytidine on the phosphorylation and cytotoxicity of arabinosyl nucleosides.. Cancer Res.

[OCR_00661] Grunewald R., Abbruzzese J. L., Tarassoff P., Plunkett W. (1991). Saturation of 2',2'-difluorodeoxycytidine 5'-triphosphate accumulation by mononuclear cells during a phase I trial of gemcitabine.. Cancer Chemother Pharmacol.

[OCR_00669] Grunewald R., Kantarjian H., Du M., Faucher K., Tarassoff P., Plunkett W. (1992). Gemcitabine in leukemia: a phase I clinical, plasma, and cellular pharmacology study.. J Clin Oncol.

[OCR_00673] Grunewald R., Kantarjian H., Keating M. J., Abbruzzese J., Tarassoff P., Plunkett W. (1990). Pharmacologically directed design of the dose rate and schedule of 2',2'-difluorodeoxycytidine (Gemcitabine) administration in leukemia.. Cancer Res.

[OCR_00680] Heinemann V., Hertel L. W., Grindey G. B., Plunkett W. (1988). Comparison of the cellular pharmacokinetics and toxicity of 2',2'-difluorodeoxycytidine and 1-beta-D-arabinofuranosylcytosine.. Cancer Res.

[OCR_00686] Hertel L. W., Boder G. B., Kroin J. S., Rinzel S. M., Poore G. A., Todd G. C., Grindey G. B. (1990). Evaluation of the antitumor activity of gemcitabine (2',2'-difluoro-2'-deoxycytidine).. Cancer Res.

[OCR_00692] Lund B., Kristjansen P. E., Hansen H. H. (1993). Clinical and preclinical activity of 2',2'-difluorodeoxycytidine (gemcitabine).. Cancer Treat Rev.

[OCR_00697] Mattern J., Bak M., Hahn E. W., Volm M. (1988). Human tumor xenografts as model for drug testing.. Cancer Metastasis Rev.

[OCR_00702] Molthoff C. F., Calame J. J., Pinedo H. M., Boven E. (1991). Human ovarian cancer xenografts in nude mice: characterization and analysis of antigen expression.. Int J Cancer.

[OCR_00710] Ruiz van Haperen V. W., Veerman G., Noordhuis P., Vermorken J. B., Peters G. J. (1991). Concentration and time dependent growth inhibition and metabolism in vitro by 2',2'-difluoro-deoxycytidine (gemcitabine).. Adv Exp Med Biol.

[OCR_00715] Winograd B., Boven E., Lobbezoo M. W., Pinedo H. M. (1987). Human tumor xenografts in the nude mouse and their value as test models in anticancer drug development (review).. In Vivo.

